# Artificial, Triple-Layered, Nanomembranous Wound Patch for Potential Diabetic Foot Ulcer Intervention

**DOI:** 10.3390/ma11112128

**Published:** 2018-10-29

**Authors:** Mostafa Mabrouk, Pradeep Kumar, Yahya E. Choonara, Lisa C. du Toit, Viness Pillay

**Affiliations:** 1 Wits Advanced Drug Delivery Platform Research Unit, Department of Pharmacy and Pharmacology, School of Therapeutics, Faculty of Health Sciences, University of the Witwatersrand, Johannesburg, 7 York Road, Parktown 2109, South Africa; mostafamabrouk.nrc@gmail.com (M.M.); pradeep.kumar@wits.ac.za (P.K.); yahya.choonara@wits.ac.za (Y.E.C.); lisa.dutoit1@wits.ac.za (L.C.d.T); 2 Refractories, Ceramics and Building Materials Department, National Research Centre, 33 El Bohouthst. (former EL Tahrirst.), Dokki, Giza P.O.12622, Egypt

**Keywords:** diabetic foot ulcer, membranousfiber patch, mechanical properties, electrospinning, polymers, thin films

## Abstract

The present work aims to electrospin a triple layered wound patch for potential treatment of diabetic foot ulcers (DFU). The patch consisted of poly(acrylic acid) (PAA) as the skin contacting layer, polyvinyl pyrrolidone (PVP) as the middle layer, and polycaprolactone (PCL) as the outermost layer, wherein the PVP layer was loaded in situ with an antibiotic (ciprofloxacin, CFX). Morphology and mechanical properties were investigated using SEM and texture analysis. Patch quality was studied with regards to wettability, adherence, water resistance, and moisture uptake of individual layers. SEM results confirmed the fibrous and membranous nature of layers with a nano-to-micro size range. Mechanical properties of the composite patch demonstrated a tensile strength of 12.8 ± 0.5 MPa, deformation energy of 54.35 ± 0.1 J/m^3^, and resilience of 17.8 ± 0.7%, which were superior compared to individual layers. Patch quality tests revealed that the PCL layer showed very low wettability, adherence, and moisture uptake compared to the PVP and PAA layers. In vitro drug release data revealed an increase in cumulative drug release with higher drug loading. The results above confirm the potential of a triple layered, tripolymeric, wound patch for DFU intervention.

## 1. Introduction

Diabetes complications include peripheral neuropathy, vascular diseases, retinopathy, nephropathy, and immune suppression [1]. Diabetic foot ulcers (DFU) are a major complication of diabetes which affect over 15% of people living with this disease [2]. This is mainly due to the lack of adherence to treatment and poor foot care. DFU can cause soft tissue and bone infections, generally due to an exposed wound site and immuno-suppression [3]. The current treatment protocol for DFU includes antibiotics, antiseptics, debridement of the wound, and wound dressing—all crucial for wound care and healing [4]. Membranous delivery systems have been used since the 1970s for localized and prolonged drug delivery to the dermis with improved compliance due to reduced dosing frequency. Available wound dressings possess certain disadvantages such as possibility of local irritation at the site of application, erythema, itching, local edema, and allergic reactions [5].

Several synthetic polymers have been employed in the fabrication of nanofibrous patches for treatment of DFU or as a wound dressing. These include poly(lactic-co-glycolic acid) (PLGA), poly(lactic acid), and PCL. These polymers represent an important class of biodegradable synthetic polymer and are readily employed for wound dressing, owing to their mechanical strength and relative biocompatibility [6,7,8,9,10,11]. Recently, our research team has produced antibacterial wound dressings based on poly(vinyl alcohol) (PVA) hybridized with calcium silicate [12]. However, classical usage of electrospun nanofibers for this application was only directed to the single layer patch, which would affect the expected life time for those patches. Therefore, we envisage prolonging the life time of the patch and extending its healing effect through fabrication of a multi-layered patch. 

In this work, to overcome disadvantages of available wound dressings, a triple layered nanofiber patch was designed, employing three individual electrospun layers comprising poly(acrylic acid) (PAA), polyvinyl pyrrolidone (PVP), and polycaprolactone (PCL). Due to its high hydrophilicity, PAA was selected to form the first layer which will be in direct contact with the DFU, aid with good adherence of the patch, and potentially enhance moisture uptake. The second layer was prepared from PVP incorporated with ciprofloxacin, as it has a broad spectrum of activity against Gram negative and Gram positive bacteria commonly found in DFUs. Finally, a hydrophobic polymer PCL was selected to be the third and outermost layer in order to prevent water penetration through the patch.

## 2. Materials and Methods

### 2.1. Patch Fabrication, Morphological, and Mechanical Characterizations

PAA (MW 450,000 g/mol), PCL (MW 80,000 g/mol), PVP (MW 400,000 g/mol), and ciprofloxacin (≥98.0%) were obtained from Sigma Aldrich, St. Louis, MO, USA. Nanofibers were electrospun using a NANOSPINNER24 (Inovenso Ltd, Co., Istanbul, Turkey) with equipment variables set as following: 4-head nozzle, collector speed 400 rpm, axial movement 56 mm, distance between collector and spin nozzle 180 mm, and 23–25 V of applied voltage. Firstly, PAA solution (7% *w*/*v*) was prepared in ethanol and pumped at a rate of 17 mL/h. Secondly, 20% *w*/*v* PVP polymer was prepared in ethanol and CFX was loaded in situ into PVP solutions with various drug polymer ratios of 1:10, 1:20, and 1:30. CFX-loaded solutions were pumped at a rate of 7 mL/h. Finally, 10% *w*/*v* PCL solution prepared in dichloromethane was pumped at a rate of 7 mL/h to complete patch fabrication. Surface morphology and mechanical properties of nanomembranous fibers were determined using a bench-top PhenomTM SEM (FEI Company, OR, USA) and a Textural Analyzer (TA.XTplus Texture Analyzer Stable Microsystems, Surrey, England). 

### 2.2. Patch Quality Tests

Patch quality was studied through investigation of wettability, adherence, water resistance, and moisture uptake of each layer of the patch. In addition, in vitro drug release behavior was conducted in PBS over 48 h and determined using a UV spectrophotometer.

#### 2.2.1. Wettability

Wettability of patches was determined using the water break test. Practically, 2 cm × 2 cm pieces of each layer of the patch were cut and placed on a flat surface. Afterwards, a few drops of water were poured onto each layer to observe distribution of water on the surface. Higher water distribution indicates good wettability and lower water distribution indicates poor wettability.

#### 2.2.2. Adherence Test

The adherence of each layer was determined using the thumb tack test. In detail, a thumb was pressed onto each layer (2 cm × 2 cm) of the patch for about 5 s and then quickly withdrawn. This test gives a prediction of adhesive behavior with the skin.

#### 2.2.3. Water Resistance

The water resistance test was conducted in simulated body fluid (SBF), briefly, 2 cm × 2 cm pieces of each layer were submersed into 100 mL of SBF solution. Containers were then incubated for 7 days. Thereafter, solutions were filtered using 125 mm filter papers. Precipitates were then weighed to determine *WAC* and *WR* according to Equations (1) and (2):

(1) $$WAC=\frac{W_{2}-W_{1}}{W_{1}}\times 100.$$

(2)  WR=100−WAC. 

#### 2.2.4. Moisture Uptake

Moisture uptake of each layer of the prepared patch was determined as follows: Nanofiber patches were prepared with dimensions of 1 cm × 1 cm and weighed, weighed patches were kept in a desiccator containing a saturated solution of potassium chloride at room temperature for 24 h, thereafter nanofiber patches were reweighed and percentage of moisture uptake was calculated using Equation (3):

(3) $$\%\;\mathrm{Moisture}\;\mathrm{uptake}\;=\frac{W\;-W_{0}}{W_{0}}\times 100.$$

where *W* is the final weight and *W*_0_ is the initial weight.

#### 2.2.5. In Vitro Drug Release

In vitro drug release was conducted in phosphate buffer saline (PBS), briefly, 2 cm × 2 cm pieces of each layer were submersed into 50 mL of PBS solution at 37 °C. Containers were then incubated for 48 h. Thereafter, 3 mL of PBS solution was withdrawn and replaced with fresh PBS to maintain the concentration gradient. CFX concentration in the PBS solution was determined at 277 nm using UV-Vis spectroscopy (Lambda 25 UV/Vis Spectrophotometer, PerkinElmer, Waltham, MA, USA). 

## 3. Results and Discussion

### 3.1. Morphological and Mechanical Properties

**Figure 1:**
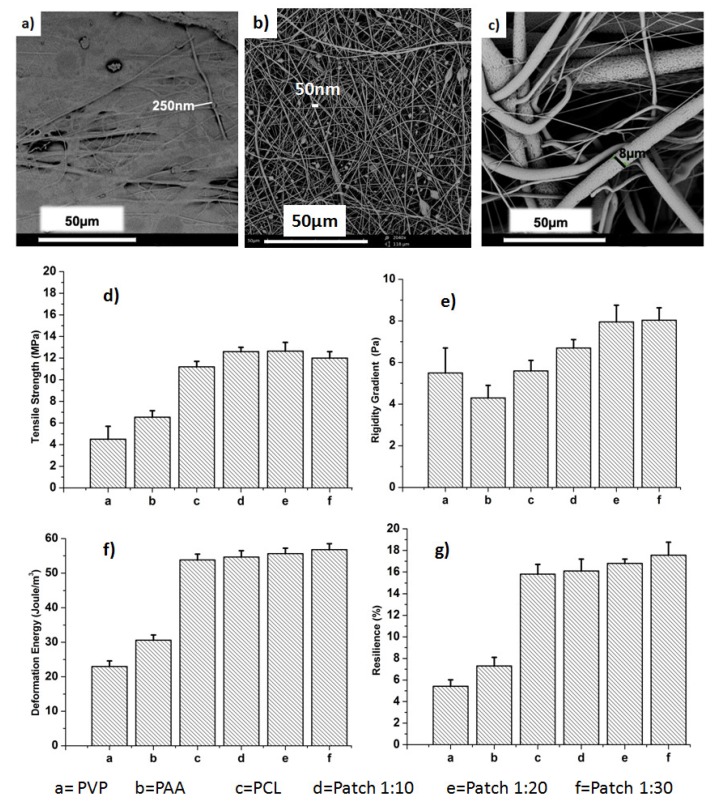
SEM images of (**a**) poly(acrylic acid) (PAA) membranous fibers; (**b**) ciprofloxacin (CFX) loaded polyvinyl pyrrolidone (PVP) fibers (1CFX:10PVP); (**c**) polycaprolactone (PCL) and mechanical properties; (**d**) tensile strength; (**e**) rigidity gradient; (**f**) deformation energy; (**g**) resilience (%) of membranous fibers.

PVP loaded with CFX demonstrated fine fibers in the nano-range around 50 nm, on the other hand PAA fibers were found to be in the submicron scale around 250 nm (Figure 1a,b). Nano-sized fibers are more favorable for adhesiveness and drug transfer and release through these two layers [13,14]. Drug loading concentrations are expected to demonstrate a notable effect on morphology of PVP nanofibers. However, higher drug concentrations for CFX-loaded PVP nanofibers were selected to be imaged by SEM. Furthermore, CFX entrapment within PVP nanofibers was confirmed by the presence of drug particles (Figure 1b). However, PCL fibers (Figure 1c) exhibited random orientation with different diameters due to fast evaporation of the DCM solvent [14].

Physicomechanical properties provide essential information about membranous systems, such as resistance to damage during storage and usage [15]. Mechanical properties of all investigated patches increased compared with native individual layers. In particular, tensile strength, deformation energy, rigidity gradient, and matrix resilience (%) increased from 4.1 ± 0.2 MPa, 5.5 ± 0.4 Pa, 23.0 ± 0.3 J/m^3^, and 5.2 ± 0.2% to 12.8 ± 0.5 MPa, 7.8 ± 0.2 Pa, 54.3 ± 0.1 J/m^3^, and 17.2 ± 0.7% respectively (Figure 1d–g). Presence of the PCL layer could have enhanced mechanical properties of final prepared patches and may help in maintaining their integrity when applied to the skin [16].

### 3.2. Patch Quality Tests

#### 3.2.1. Wettability Test

**Figure 2:**
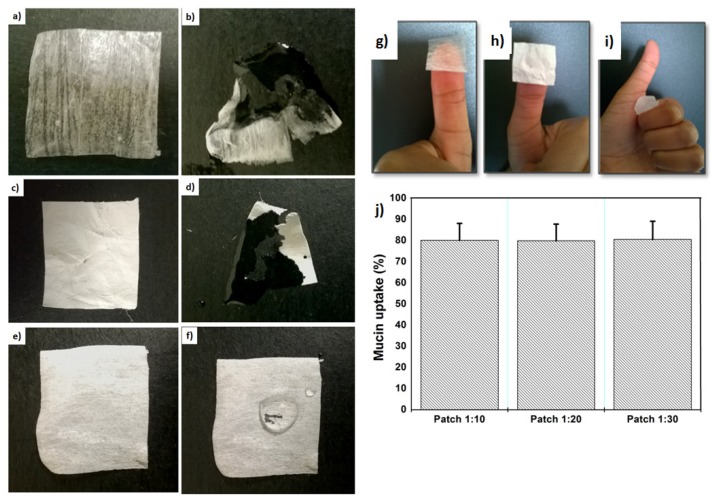
Images showing (**a**) PAA before wetting; (**b**) PAA after wetting; (**c**) PVP before wetting; (**d**) PVP after wetting; (**e**) PCL before wetting; (**f**) PCL after wetting and thumb test of (**g**) PAA; (**h**) PVP\CFX; (**i**) PCL; (**j**) mucin uptake (%) of the patches.

Wettability describes the preference of a solid to be in contact with one fluid rather than another, based on the balance of surface and interfacial forces [17]. PAA and PVP layers exhibited very high wettability due to their higher hydrophilicity (Figure 2). Good wettability of these layers may enhance drug release through patch layers [18]. Conversely, the PCL layer exhibited a very low (non-significant) wettability (Figure 2f) due to its higher hydrophobicity [19].

#### 3.2.2. Adherence and Mucoadhesion Tests

The thumb test is a unique test because it does not require heat or chemical treatment in order to acquire relatively good adhesive strength to a wide range of substrates [20]. Results of the thumb test indicated that PAA and PVP layers have great potential as an adhesive for a dermatological patch and have comparable performance (Figure 2g,h). Conversely, the PCL layer displayed no adherence, as it did not adhere to the thumb. This confirms the reliability of selecting PCL as the outer layer of this triple patch, as it prevents water penetrating the wound through the patch and will allow the patch to stay on the wound site for longer. Moreover, mucin uptake (%) confirmed that drug concentration has no effect on adhesion properties, as all patches exhibited the same amount of mucin uptake (Figure 2j).

#### 3.2.3. Water Resistance

**Figure 3:**
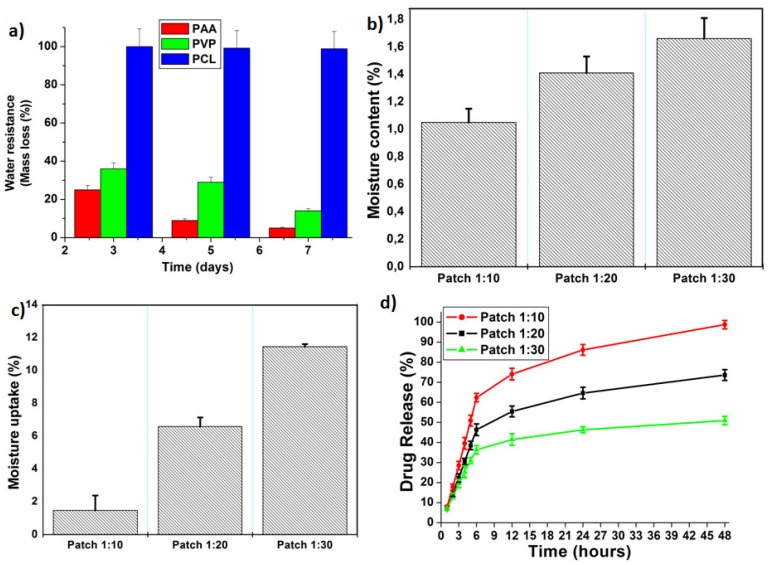
Profiles showing (**a**) water resistance (%) of individual layers of the patch after immersion in simulated body fluid (SBF) for different time intervals; (**b**) moisture content (%); (**c**) moisture uptake (%); (**d**) CFX release from prepared patches.

The water resistance (mass loss) test was conducted over seven days of incubating individual patch layers in SBF. The PCL layer possessed higher water resistance (98%) after seven days of immersion (Figure 3a). On the other hand, PAA and PVP layers exhibited the lowest water resistance (22%) even after three days. It is worth noting that a good patch for the treatment of DFU should possess high water resistance from its outer layer, as well as low water resistance from skin contacting layers.

#### 3.2.4. Moisture Content

Moisture content of the patches (1:10, 1:20, and 1:30) was 1.05%, 1.41%, and 1.66% respectively (Figure 3b). This result revealed that the highest moisture content was observed for the lowest drug content. This may be because the drug content increment decreased porosity (%) of the patch, which in turn would decrease moisture content (%) of the patch. A low moisture content (%) helps the patch to remain stable and prevents it from being completely dry and brittle during storage, thus maintaining its mechanical integrity.

#### 3.2.5. Moisture Uptake

Lower percentages of moisture uptake are preferred for wound dressings as this will protect the patch against microbial contamination for a long time. Moisture uptake of samples ranged within 1.47–11.44%. Increases in moisture uptake may be attributed to the hygroscopic nature of PAA and PVP polymers. Additionally, results revealed that the highest moisture uptake was observed for the lowest drug content. The decrease in drug content may be accompanied with increased porosity, which in turn would increase moisture uptake of the patch. 

#### 3.2.6. Drug Content and Drug Release

Drug content of nanofibers patches was approximately 83.7 ± 5.1% of the starting drug concentration (Figure 3d). Interestingly, all patches demonstrated a prolonged burst release of 30% to 60% within the first 6 h and thereafter the release pattern was linear, up to 48 h. These results could be explained by the fact that CFX was loaded into PVP nanofibers through physical blending (confirmed by SEM image (Figure 1b)) and some of the loaded CFX appears entrapped within the mesh structure outside the nanofibers. Therefore, the drug may have partially adsorbed onto PVP nanofibers. Once exposed to PBS media, physical attachments between CFX and PVP nanofibers were easily disentangled due to hydrophilicity of PVP and PAA layers [18]. This led to a significant initial release phase followed by linear behavior of the entrapped drug up to 48 h. Furthermore, it is worth noting that drug release percentage increased with drug content increment. It is worthy to highlight that topical antibiotic treatment has the advantage of minimizing the effective dose needed to inhibit bacterial infection and limit any systemic effects of the drug [21]. However, delivery of excess antibiotic could result in delayed healing due to development of antibiotic resistance. In view of this, 48 h of CFX administration with high frequent doses would be sufficient to treat bacterial infection. Moreover, the CFX-PVP nanofiber interface is clearly noted in SEM images of CFX-loaded nanofibers. This interface was originated through blending CFX within the PVP polymer matrix. According to previous studies [22,23,24], it is well known that miscible blends can help to regulate release behavior by changing the polymer/drug ratio, which can be seen in this case and resulted in various polymer-drug interactions or interfaces, which in turn modulate bioactive release behavior. Some studies have explained this behavior by suggesting the formation of a homogeneous matrix with the drug randomly distributed throughout the polymer structure at low loading and a heterogeneous matrix at high drug loading. Results revealed that nanofiber patches increased drug release (%) due to the PVP nanostructure.

## 4. Conclusions

Electrospun nanofibers were used to design new wound dressings loaded with different concentrations of CFX for local treatment of DFU. Prepared patches showed enhanced mechanical properties compared to native individual layers. The in vitro drug release study indicated an initial surge followed by consistent controlled release for a minimum of 48 h. Based on patch quality tests, designed CFX-loaded, PAA/PVP/PCL triple layer patches are suitable for potential diabetic foot ulcer treatment. The current design of the triple layer patch is expected to represent an alternative to the classical single layer patch, owing to its exceptional properties. Furthermore, in vivo studies and clinical studies will be conducted to further understand how this product can be delivered to the market in a low cost and effective form. The concept of using a triple layer patch for treatment of diabetic foot ulcers was explored in this research to facilitate treatment by offering a single-stage procedure.
